# Study protocol for a pilot quasi-experimental study on oral health education for nurses and community health workers in Nigeria

**DOI:** 10.3389/fpubh.2024.1398869

**Published:** 2024-06-07

**Authors:** Abimbola M. Oladayo, Folake B. Lawal, Oyinkansola O. Sofola, Omolara G. Uti, Afolabi Oyapero, Adetayo Aborisade, Bernal Stewart, Carlo Amorin Daep, Deon Hines, Jacinto Beard, Aderonke Dedeke, Omotayo F. Fagbule, Adeola T. Williams, Obioma C. Uchendu, Kudirat Ohiare, Adetomiwa O. Adedire, Abdul-Kabir Adegoke Yahya-Imam, Oluwagbenga Ilori Adeniji, Aishatu Baba Mele, Amina Sani Baffa, Ifeoluwa Adetula, Taiwo A. Lawal, Gbemisola Aderemi Oke, Azeez Butali

**Affiliations:** ^1^Department of Oral Pathology, Radiology and Medicine, College of Dentistry, University of Iowa, Iowa City, IA, United States; ^2^Iowa Institute for Oral Health Research, University of Iowa, Iowa City, IA, United States; ^3^Department of Periodontology and Community Dentistry, Faculty of Dentistry, College of Medicine, University of Ibadan, Ibadan, Oyo, Nigeria; ^4^University College Hospital (UCH), Ibadan, Oyo, Nigeria; ^5^Department of Preventive Dentistry, Lagos University Teaching Hospital, Idi–Araba, Lagos, Nigeria; ^6^Department of Preventive Dentistry, Faculty of Dentistry, Lagos State University College of Medicine, Lagos, Nigeria; ^7^Department of Oral Diagnostic Sciences, Faculty of Dentistry, Bayero University Kano, Kano, Nigeria; ^8^Department of Oral Diagnostic Sciences, Aminu Kano Teaching Hospital, Kano, Nigeria; ^9^Colgate-Palmolive Company, Piscataway, NJ, United States; ^10^National Dental Association Foundation, Washington, DC, United States; ^11^Department of Child Oral Health, Faculty of Dentistry, College of Medicine, University of Ibadan, Ibadan, Oyo, Nigeria; ^12^Department of Community Medicine, University of Ibadan, Ibadan, Oyo, Nigeria; ^13^College of Dentistry, Lagos University Teaching Hospital, Idi–Araba, Lagos, Nigeria; ^14^Department of Oral and Maxillofacial Surgery, Lagos University Teaching Hospital, Idi–Araba, Lagos, Nigeria; ^15^Department of Restorative Dentistry, Lagos University Teaching Hospital, Idi–Araba, Lagos, Nigeria; ^16^Department of Preventive Dentistry, Aminu Kano Teaching Hospital, Kano, Nigeria; ^17^Division of Pediatric Surgery, Department of Surgery, University of Ibadan and University College Hospital, Ibadan, Oyo, Nigeria

**Keywords:** primary health care (PHC), oral health education, oral health, nurses, community health workers, pilot study

## Abstract

**Introduction:**

The primary health care system provides an ideal setting for the integration of oral health into general health care as well as equitable access to oral health care. However, the limited oral health knowledge of primary health care workers necessitates appropriate training before they can participate in health promotion efforts. This pilot training was designed to examine the impact of the Oral Health Education module for Nurses and Community Health Care Workers on their oral health awareness and referral practices.

**Methods:**

This study will utilize a quasi-experimental design (pre-and post with a non-equivalent control group) to assess the impact of a five-day pilot oral health education program on the knowledge and referral practices of Nurses and Community Health Workers in primary health care centers in three states in Nigeria-(Lagos, Oyo, and Kano). The training modules were developed based on the six iterative steps described in the intervention mapping framework – needs assessment, highlighting program objectives and outcomes, selection of theory and mode of intervention, designing program based on theory, designing implementation plans, and developing an evaluation plan. Only the intervention group will participate in the full educational training sessions but both groups will complete the pre-and post-intervention questionnaires.

**Discussion:**

This pilot training combined the standardized training modules from the recently launched “*Oral Health Training Course for Community Health Workers in Africa”* and a newly developed maternal and child oral health module by our group using an evidence-based approach. To the best of our knowledge, this is the first program to examine the impact of the standardized OpenWHO modules. The success of this training will lay the foundation for developing a sustained channel for providing oral health education at the primary health care level in Nigeria, West Africa, and Africa.

## Introduction

Oral diseases and conditions are the most common non-communicable diseases (NCDs) and are increasingly becoming a global public health concern. Despite being largely preventable, about 50% of the global population (~3.5 billion) have some form of oral disease condition (1, 2). In 2019, about 44% of the population in the World Health Organization (WHO) African Region was affected by oral diseases. This data reflects the highest increase in the prevalence of oral conditions across the world over the past three decades (2). Regarding the burden of oral diseases in Nigeria, the 2019 estimates provided by the WHO reported a prevalence of 35.5% for deciduous caries (aged 1–9 years), 23.9% for decay on permanent teeth (aged ≥5 years), 25.1% for severe periodontal disease (aged ≥15 years); 7.9% for edentulism (aged ≥60 years) and a 1.2% age-standardized incidence rate of lip and oral cancer (2).

Adopting a preventive approach to oral health and integrating oral health into general health care is now a global health priority and is essential to achieving a broader impact on the community and population level and cost-effectiveness, especially in settings with limited healthcare resources (2–5). Access to preventive interventions, early disease detection and management, and more equitable access to comprehensive, high-quality care are just a few possible benefits of oral health-primary care integration (6, 7). However, many Nigerians do not have access to preventive dental care; as such, it remains an unmet health need (3).

In Nigeria, there is persistent difficulty in accessing oral care and inequalities in oral health outcomes as the oral health care system is characterized by limited resources, an overstretched workforce, high out-of-pocket expenses, the predominance of a private dental service model and little or no access for rural communities (8–14). Despite the appearance of progress in oral health-related matters, as evidenced by the introduction of the 2012 National Oral Health Policy (NOHP), which highlighted the role of PHC workers in integrating oral health with general health, efforts made to implement this comprehensively have failed due to limited resources (15). Furthermore, the persistent shortage and current decline in the Nigerian oral health workforce (1 dentist to 40,000 people) highlight the ongoing challenge of inadequate personnel in this country (9) and further bolsters the need to utilize and train midlevel and allied health workers to share and shift the current task burden (2, 8, 16).

In the present day, Nigerian PHCs rarely or sparingly provide oral health care (15). The direct connection of the PHCs to communities and individuals makes it the ideal platform for reaching the “*last mile*” population, primarily found in rural and underserved areas (7, 15, 17). PHC staff, including community health workers and nurses, are trusted members of the community. Exposing them to oral health education would provide them with a channel to amplify accurate health information within their communities while improving awareness about the burden of oral diseases. This could help promote health and oral health-seeking behaviors as well as prioritization of interventions for prevention and management. It is thus essential to adequately educate PHC workers before enrolling them on oral health promotion efforts, since research has revealed a lack of exposure to oral health education via their traditional training (18). Furthermore, successes from community-driven oral health promotion practices in Africa have been reported in countries such as Burkina Faso, Kenya, Cameroun, and Gambia (7). However, the absence of a model upon which oral health promotion can be built is a significant barrier to program implementation (18). This is because the application of sound and evidence-based models and theories provide a foundation for program planners to design, implement and evaluate effective health promotion programs (19). One such approach is Intervention Mapping (IM). IM is a planning framework useful for interventions targeted at behavioral change (20). It provides a template for the systematic planning, development, and evaluation of health promotion interventions (21, 22). IM has been characterized by three perspectives; the use of empirical evidence and theories, an ecological approach and stakeholder participation (20, 21), the outcome of which is more effective interventions (22). Despite its use in developing and implementing health interventions across the globe, there is a paucity of information on its use in health promotion planning in Nigeria, particularly in relation to oral health promotion.

The recently launched OpenWHO oral health promotion course for community health workers in Africa provides an opportunity to train PHC workers with standardized training modules, promoting consistency among trainees while serving as a foundation for improvement in the future (23). Moreover, a focus on vulnerable population groups such as infants, young children and pregnant women, who are usually disproportionately affected by gaps in oral health care (24, 25) via increased awareness and training on maternal and child oral health (MCOH) will allow for the provision of culturally appropriate health information for common oral diseases. These oral diseases include debilitating conditions such as orofacial cleft and Noma and training PHC workers will help foster community support and end the stigmatization associated with these conditions. Based on the six iterative steps described in the IM framework, this paper presents the protocol for Project OHE-NCHeW (oral health education module for Nurses and Community Health Care Workers).

## Methods

### Program development using the IM framework

**Figure 1:**
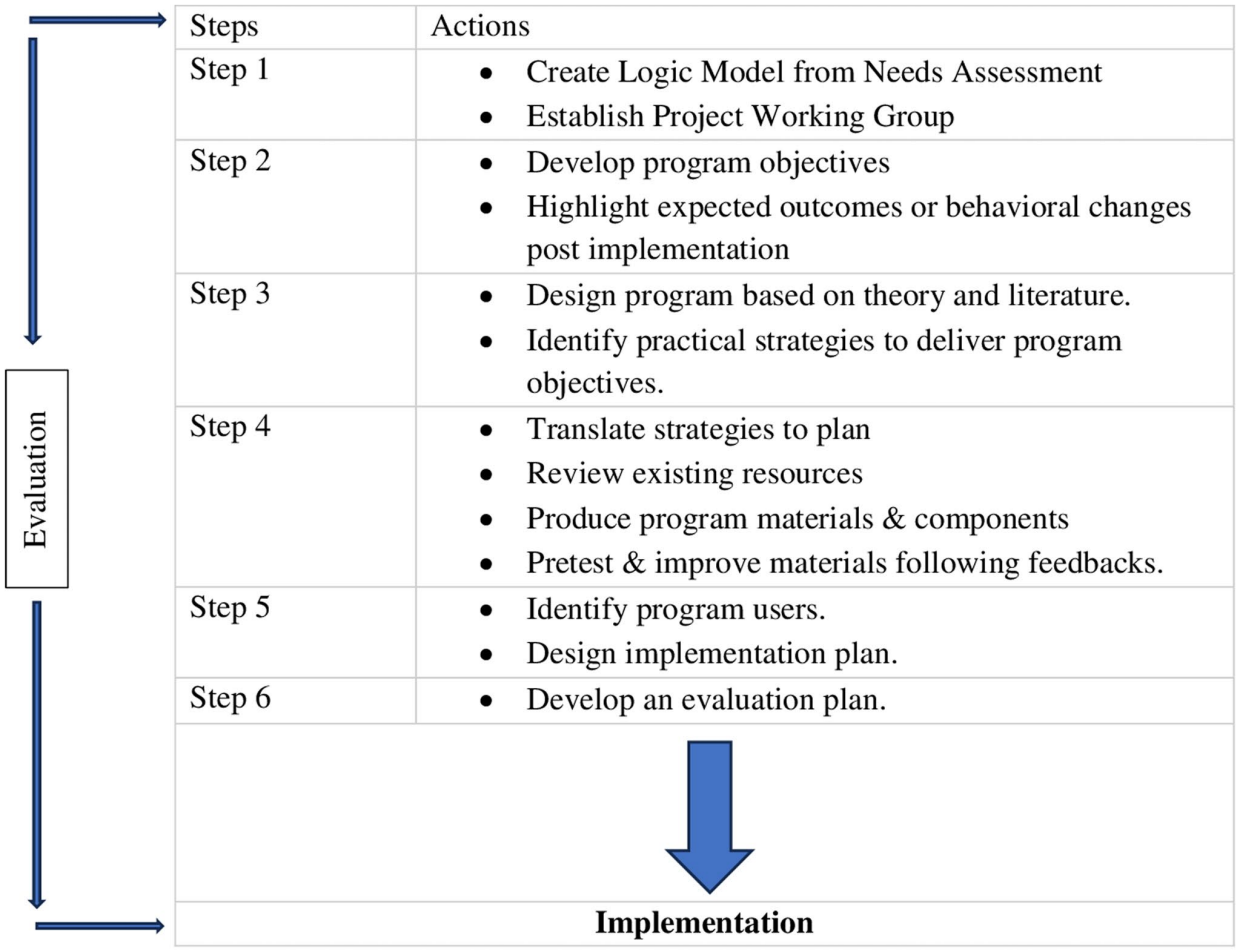
Intervention mapping steps adapted from planning health promotion programs (21).

The week-long educational intervention was designed using the iterative steps of the IM framework (Figure 1). Step 1 involved conducting a literature review by searching public databases for existing peer-reviewed literature, policy documents and organizational reports to explore the barriers and inequities in accessing quality and comprehensive oral care in developing countries such as Nigeria. Specific focus was placed on the integration of oral health into general health care services. The opinions of key stakeholders consisting of a multidisciplinary team of experts at different levels of the health care system, including dentists and dental public health practitioners, public health practitioners (with vast experience in oral health education, promotion and programming) (26–30), nursing educators, community health educators, industry partners, policymakers, nurses, and community health workers were also sought to answer these questions. Oral health integration, specifically in the PHC system, has been described as an efficient and cost-effective way of promoting population oral health in developing countries (2, 3, 8, 30, 31). The information obtained from the knowledge gaps identified in the literature and stakeholder engagement guided the development of program objectives and outcomes using a logic model in step 2 (Table 1).

**Table 1:** Logic Model.

Resources	Activities	Outputs	Short & Long Term Outcomes
Project Working Group. Time for planning and implementation. Project Funding from Industry Partners (Colgate-Palmolive and National Dental Association Foundation). Training Materials. Partners at Dental Colleges and State Department of Health.	Mobile *PHC workers and community support. Design training and workshop materials. Implement training strategy. Promote community oral health education. Create an evaluation plan.	# of *CHWs & Nurses trained. # of *OHE sessions completed/3months. # of PHC patients who have received OHE/3 months. # of patients referred to dental centers for oral conditions/three months.	**Short term** Increased *OH knowledge and awareness among participants. Change in patient attitude about the need for oral health care. Increased # of referrals to dental centres. **Long term** Increased competency among PHC workers in providing OHE. Incorporation of OHE into the training curriculum of PHC workers. Increased use of preventive oral care services. Decreased prevalence of oral health disease conditions.
**Assumptions** Limited oral health training restricts the ability of PHC workers to provide adequate oral hygiene education. To start this process, those on ground would require some assistance to start. PHC workers and other stakeholders are interested in and fully supportive of the project.		**External Factors** Continuation of funding for training activities. Availability of time for PHC workers to incorporate OHE into health promotion activities.	

*PHC, Primary Health Care; OH, Oral Health; OHE, Oral Health Education; CHW, Community Health Worker.

In step 3, we identified an evidence-based approach most likely to influence change using the Lay Health Worker Model/Promotora de Salud (32), a public health model of workforce development targeted at health professionals such as community health workers, in this context, who lack formal oral health training but are members of the communities that they work in (33, 34). This model is often utilized to improve and support access to care in settings with workforce shortages (35). Although this is not commonly used in this population, the promising outcomes reported as it concerns oral health-related knowledge and behaviors in minority populations (36–39) and the need for a more competent allied health workforce capable of providing culturally appropriate oral health care in Nigeria makes it applicable to this study. By exploring parallel processes through collaborative partnerships and considering cultural nuances in the curation of training materials to maximize relevance and acceptability, we anticipate an impactful and lasting program outcome. This led to the fourth step, where program components, like training modules, assessment questions, activity sheet and the logbook for evaluation, were designed following extensive deliberation with all stakeholders. For the fifth and sixth steps, we established how, when, and where the training will be implemented and developed an evaluation plan to assess the effectiveness of the program. In this case, the impact of OHE-NCHeW will be immediately evaluated post-intervention and after 3 months in the training and intervention group.

### Study objective

This study aims to report the experience of adapting, developing, facilitating, and evaluating a five-day pilot of the modules for Project OHE-NCHeW in three states in Nigeria. This training seeks to examine the impact of the standardized OpenWHO oral health promotion modules and an additional Maternal and Child Oral Health (MCOH) module on participants’ oral health knowledge and referral practices as they relate to oral health. This pilot is intended to serve as a foundation for promoting oral health knowledge among primary care workers in the country. Our long-term objective with this systems-level intervention is to increase the capacity of PHC workers (Nurses and Community Health Workers) to provide oral health promotion via oral health education and engage patients attending PHCs to promote primary prevention methods for oral health in a bid to reduce risk for oral diseases.

### Research question

Does the introduction of oral health education modules by designated trainers improve the oral health knowledge, attitude, and referral practices regarding oral conditions in PHC Nurses and CHWs?

#### Null hypothesis

No measurable difference will be observed in the post-intervention knowledge scores and referral practices as it relates to oral health between participants with access to the 5-day training workshop vs. the control group.

#### Alternate hypothesis

An observable difference will be present in post-intervention knowledge scores and referral practices as it relates to oral health between participants with access to the training workshop vs. the control group.

### Study design

We will utilize a quasi-experimental design (QED) (pre-and post-with a nonequivalent control group) to assess the impact of a five-day pilot oral health education program on the knowledge level and referral practices of Nurses and Community Health Workers in PHCs in three States in Nigeria - (Lagos, Oyo and Kano). A three-month post-training evaluation will follow this. A pre-training evaluation will be conducted prior to the commencement of training, and a post-evaluation will be conducted immediately and 3 months after training. Furthermore, we have included a qualitative assessment via unstructured interviews with the program trainers to explore their experience delivering the training modules and interacting with participants. We will also conduct a focus group discussion (FGD) with the participants in the intervention group to have an in-depth understanding of their opinions, training impact and points of improvement at the 3-month post-training evaluation. Quasi-experiments are often conducted to assess the effectiveness of a treatment, such as educational intervention in field settings where random assignment is not feasible (40–42). This method remains a quick and convenient approach to evaluating a target group to which an intervention has been applied (43).

### Sample size estimation

For the feasibility of pilot studies, research has shown that a sample size consisting of 30 participants in a group is sufficient (44, 45). Based on this evidence, we plan to recruit a minimum of 10 and a maximum of 20 nurses and community health workers in each group per study site. Overall, the estimated sample size for the quantitative component of this pilot study across the three study sites is 120 (60 participants in the intervention group and 60 participants in the control group). The data obtained from this pilot program will be used to estimate a suitable sample size for our major study in the future (46). For the qualitative assessment, unstructured interviews will be conducted with the trainers. A full-group focus group discussion [>7 participants per group as defined by Cortini et al. (47)] will be conducted at each study site. For this study, we intend to engage 8–10 participants per site.

### Participants

For each study site, 40 participants (20 interventions and 20 controls) will be selected from a minimum of 4 PHCs. Participant selection will be by cluster sampling in each study site. In order to prevent cross-contamination among the participants, those in the intervention and control groups will be selected from different centers. Eligible participants will be 18 years and older, who speak and respond to questions in English, are employed full-time as a Nurse or CHW at the selected PHCs, have had no previous exposure to oral health training, and be willing to participate in the study. Participants with previous exposure to oral health training and those who cannot commit to the entire duration of the training will be excluded from the study.

### Participant recruitment

The permission to conduct the study was sought, and relevant approvals were obtained from the Permanent Secretary (PS) of the State Primary Boards, Permanent Secretaries of State Health Districts, Director of Nursing Services and the Director of Community Services. Nurses who are in the dental and maxillofacial unit and/or nurses who have assisted with Smile Train education and surgeries were excluded. The nominal rolls of nurses and community health workers in the State Health Districts served as the sampling frame, and five nurses and five community health workers who met the selection criteria were selected from each district by simple random sampling (balloting). To avoid contamination, participants from Health districts who were from more distant locations were selected for the control group, while the participants from locations closer to the training center were selected for the intervention group. Following the IRB approval from the Ethics Committee of the participating centers, each recruited participant signed a written consent before active recruitment into the study.

### Intervention and control

The intervention and control groups will consist of participants selected by a non-random process but with comparable characteristics. Across the three study sites, a total of 60 PHC workers (20 Nurses and CHWs at each site) will be assigned to the intervention group, and a corresponding number of participants will constitute the control group. Participants in the control group will receive a one-off conventional oral health education session consisting of a lecture on oral health, tooth brushing, and diet and oral hygiene demonstration using dental models. The intervention group will be exposed to the week-long pilot training on oral health education delivered by designated trainers (see Table 2). They will also participate in group activities such as oral exams and activity sheets. Each module is expected to last for approximately 4 h. Participants in the intervention group (those who will be trained) will be compared to the control group at each study site. Before the training begins, each participant will be administered a pre-intervention questionnaire to evaluate their oral health knowledge at baseline. The assessment will be repeated immediately after training and after 3 months. These evaluations not only serve as a means of monitoring training impact but also allow participants to observe how they improve throughout the training.

**Table 2:** Program and module delivery for the five-day training.

Day	Module
Day 1	Pre-intervention test Introduction to oral health
Day 2	Introduction to oral diseases and conditions
Day 3	Techniques in oral health promotion and oral disease prevention
Day 4	School and community-based oral health promotion
Day 5	MCOH Post-intervention test Facilitator assessment of participants capability to provide oral health education.

The training modules will include the adaptation of existing training resources - Oral Health Training Course for Community Health Workers in Africa developed by the World Health Organization (WHO),^1^ Oral Health in Comprehensive Cleft Care Educational resources for non-oral health professionals developed by the World Dental Federation (48), and a maternal and child oral health (MCOH) module co-developed by our group consisting of subject matter experts in the field of dental education, dental public health and public health with particular emphasis on stigmatizing conditions such as orofacial clefts and Noma. Participants will be exposed to 4 modules from the OpenWHO course and an additional MCOH module (Table 2). These modules will cover an introduction to oral health, an introduction to oral diseases and conditions, techniques in oral health promotion and oral disease prevention, school and community-based oral health promotion and the MCOH module developed by our group. The OpenWHO course also has a special module that designated trainers will be required to study before the commencement of the training. For this pilot, a member of the research team (an Indigenous local) fluent in the local language at each study site (Hausa and Yoruba) will be designated to provide summaries and interpretation for complex terms. Each group of the participants will also have a designated group lead who will be fluent in both the Indigenous language and in English to also summarize each daily’s training module and direct the associated training activities of the participants. To corroborate dissemination of the training modules, participants will be picked randomly from each group to further explain each module’s concepts to ascertain profound understanding of interpreted complex terms and salient aspects of the training module.

### Program assessment/evaluation

The assessment of the study outcome will be performed using a quantitative and a qualitative approach. Quantitative assessment will be conducted using questionnaires administered by the designated trainers, and the data will be fed into the Qualtrics software for data management. Before the training commences, all participants will take the pre-intervention test that measures their initial knowledge of oral health. The questions will be co-developed by Dental Public Health (DPH) consultants and educators on the research team based on available evidence and will be assessed for face and content validity before deployment. The questionnaire will also be pre-tested among participants who would not be involved in the study. The control group will be immediately provided with the post-intervention evaluation after the one-day lecture. After the training, participants in the training group will be assessed using the post-test. The pre and post-test scores will be compared for participants in the treatment and control groups. Participant’s capability to screen and detect oral diseases will be assessed post-intervention using picture tests.

After 3 months, a follow-up evaluation will be conducted. The number of logged referrals from the PHCs to the dental centers for oral health conditions will also be assessed for 3 months post-training based on the information logged by these providers (see Logic Model-Table 1). In addition, 5% of patients who were referred to dental centers as entered in the logbooks will be contacted via phone calls to verify their final visit to the dental centers.

The qualitative assessment to assess the impact of the training, points of improvement and the practicability of incorporation of oral health promotion activities into maternal and child health visits will be conducted with select participants in the intervention group at the 3-month evaluation using Deliberative Focus group discussions. This approach to evaluation serves to enhance program feedback through participants’ experiences and opinions. Refresher training will be conducted accordingly in month three if necessary. Data collection will be from February to May 2024.

### Data analyses

Quantitative data analysis will be conducted using SPSS version 28. Univariate analysis presenting the frequencies and percentages for categorical variables and the means and standard deviation (SD) for the continuous variables will be performed. For the bivariate analysis, t-tests and ANOVA or their nonparametric analogs (based on normality) will be conducted for within and between group associations with a *p*-value set at <0.05. For the qualitative data analysis, audio recordings obtained during data collection will be transcribed verbatim by the research team and will be analyzed by content analysis. Data will be analyzed using Nvivo12. Responses will be coded into themes and sub-themes, ensuring data saturation when no new information is discovered in data analysis. Comparison, mapping, and verification of conclusions will be conducted and arranged according to the study objectives. Prominent quotes from the participants will be selected and cited within the report.

## Discussion

Pilot studies are essential for improving the quality and efficiency of the major study (49). Thus, the success of this pilot program could help set the foundation for sustained oral health education training for primary health care workers, invariably creating a pool of qualified peer trainers for PHC workers, PHC patients, their families, and the general population. Studies have shown how misinformation, myths and cultural beliefs among individuals and community stakeholders negatively impact oral health attitudes and care-seeking behaviors in developing countries (50–54). Promoting approaches where qualified care professionals provide adequate and accurate oral health information may thus influence positive oral health practices, leading to improved oral health and overall health. The scope of the current project will encompass most of the essential components of primary oral care recommended by the WHO by leveraging the strategic positioning of the PHC system and its link with individuals, communities, and the health care system. These recommendations include “*age-friendly primary health care for oral health, maternal and child oral health care, health communication/oral health education, disease prevention methods, early detection, pain control - emergency care for oral health, continuity of oral healthcare, supportive referral systems and priority to those people most in need*” (7). The strength of the QED approach lies in its simplicity regarding data collection, ease of implementation and associated low cost (55). However, there are some limitations to this study design. Due to non-randomization of study participants, other factors related to the outcome of interest beyond the control of the researchers could potentially influence study results (56). Secondly, it is impossible to determine causality because quasi-experimental research is not an actual experiment; thus, only associations between interventions and outcomes can be made (43). Furthermore, because the treatment and control groups are nonequivalent (group assignment is by non-random selection), selection bias may exist. The presence of a nonequivalent no-treatment control in this study reduces issues such as threats to internal validity (i.e., observing the same participants over time). This is due to the assumption that the presence of similarity between the treatment and the control groups at baseline reduces the likelihood of having significant confounder differences between both groups (55, 57, 58).

In this study, we will ensure that a consistent approach is taken to select participants within and across study sites, administer evaluation surveys and follow-up (59). Additionally, we will conduct a pre-intervention assessment (allowing for the baseline comparison of the treatment and control group), an immediate post-intervention assessment, and 3 months following the training to allow for consideration of learning or attitude decay (43). Before use, the pre and post-tests will be validated for accuracy in measuring the outcomes of interest in the study. Also, tests will be scored consistently by a non-biased grader blinded to the participants and not part of the team that designed or conducted the oral health training (43). Regarding the establishment of causality, given that the intervention- (oral health education) comes before the assessment of the outcome, associations identified in QEDs meet an essential causality requirement (60).

## Conclusion

The OpenWHO online course on oral health for CHWs in WHO African Region is the first of its kind, with several modules aimed at building participant’s capacity “*on oral health promotion, oral disease prevention, and control to meet some of the unmet demand for oral health services and strengthen the oral health system through a cross-cutting approach*” (61). To the best of our knowledge, no study has been done to document the experiences of trainees and participants engaging in this course. Additionally, this training includes an extra module which will provide culturally appropriate information for maternal and child oral health in this population. Lessons learnt could be useful to strengthen adoption and implementation practices in Nigeria and across the WHO African Region.

## Ethics statement

The studies involving humans have been approved by the Institutional Review Boards at the University of Iowa (IRB-01/202312517); the University of Ibadan/University College Hospital, Ibadan (UI/EC/23/0724); the University of Lagos/Lagos University Teaching Hospital, Lagos (ADM/DSCST/HREC/APP/6454) and Bayero University Kano/Aminu Kano Teaching Hospital, Kano (NHREC/28/01/2020/AKTH/EC/3739), Nigeria. The studies were conducted in accordance with the local legislation and institutional requirements. The participants provided their written informed consent to participate in the pilot study. Written informed consent to participate in the main study will be obtained from participants.

## Author contributions

AOl: Conceptualization, Funding acquisition, Methodology, Resources, Writing – original draft, Writing – review & editing. FL: Conceptualization, Writing – review & editing, Resources, Methodology. OS: Conceptualization, Writing – review & editing, Resources, Methodology. OUt: Conceptualization, Writing – review & editing, Resources, Methodology, Visualization. AOy: Conceptualization, Writing – review & editing, Methodology, Resources. AAb: Conceptualization, Methodology, Writing – review & editing, Resources. BS: Writing – review & editing, Resources. CD: Writing – review & editing, Resources. DH: Writing – review & editing, Resources. JB: Writing – review & editing. AD: Writing – review & editing. OF: Writing – review & editing. AW: Writing – review & editing. OUc: Writing – review & editing. KO: Writing – review & editing. AAd: Writing – review & editing. Y-IA: Writing – review & editing. IO: Writing – review & editing. AM: Writing – review & editing. ABa: Writing – review & editing. IA: Writing – review & editing. TL: Writing – review & editing. GO: Writing – review & editing. ABu: Methodology, Resources, Supervision, Writing – review & editing.

## Footnotes

^1^ https://openwho.org/courses/oral-health-community-AFRO
